# Nomogram-based prediction of asparaginase-associated pancreatitis in children with acute lymphoblastic leukemia: a retrospective study

**DOI:** 10.3389/fphar.2026.1767417

**Published:** 2026-05-19

**Authors:** Xiangyu Ding, Ruihong Li, Chenhong Jia, Yile Zhao

**Affiliations:** 1 Department of Pharmacy, Hebei Children’s Hospital, Shijiazhuang, Hebei, China; 2 Hebei Provincial Clinical Research Center for Child Health and Disease, Shijiazhuang, Hebei, China

**Keywords:** asparaginase, PEG-asparaginase, pancreatitis, acute lymphoblastic leukemia, nomogram, prediction model, children, risk factors

## Abstract

**Background:**

Asparaginase is a crucial drug in acute lymphoblastic leukemia (ALL) treatment, but its use is frequently complicated by asparaginase-associated pancreatitis (AAP). Although several risk factors for AAP have been identified, no comprehensive predictive model is currently available to assess individual patient risk. We aimed to develop and validate a nomogram for AAP risk in children with ALL.

**Methods:**

We conducted a retrospective study of 352 children with ALL diagnosed at Hebei Children’s Hospital from January 2020 to June 2025, all treated according to the Chinese Children’s Leukemia Group Acute Lymphoblastic Leukemia 2018 (CCLG–ALL–2018) protocol. Patients were divided into AAP and non-AAP groups based on the 2012 Atlanta diagnostic criteria for pancreatitis. Clinical data and laboratory parameters were systematically collected. Least absolute shrinkage and selection operator (LASSO) regression was used for variable selection, followed by multivariate logistic regression to construct the prediction model. Model performance was assessed using the receiver operating characteristic (ROC) curve analysis, calibration curves, and decision curve analysis (DCA).

**Results:**

Among the 352 patients (median age 5.0 years; 56.53% boys), 36 patients (10.23%) developed AAP. Six independent risk factors were identified: history of AAP (odds ratio [OR] = 13.20, *P* < 0.001), albumin level (OR = 0.87, *P* = 0.009), aspartate aminotransferase (OR = 1.01, *P* < 0.001), total bilirubin (OR = 1.04, *P* < 0.001), blood calcium (OR = 0.01, *P* = 0.003), and blood glucose (OR = 1.74, *P* < 0.001). The nomogram model demonstrated excellent discrimination with an area under the curve (AUC) of 0.926 (95% CI: 0.874–0.978) and good calibration (Brier score = 0.012). Internal validation confirmed robust performance with a corrected AUC of 0.923 (95% CI: 0.911–0.931). DCA indicated a positive net benefit across threshold probabilities of 0.02–0.72, suggesting that the model has strong potential for clinical application.

**Conclusion:**

A nomogram incorporating six available clinical and laboratory parameters was successfully developed and validated to predict AAP risk in children with ALL. The model demonstrated excellent predictive performance and clinical applicability, providing a valuable tool for early identification of high-risk AAP patients and guiding individualized preventive strategies to optimize treatment outcomes.

## 1 Introduction

Outcomes for children with ALL have improved worldwide to >85% (Sidhu et al., 2024). Survival rates have improved among pediatric and adolescent patients as well as young adults, with 5-year overall survival (OS) rates of 89% and 61%, respectively (Inaba et al., 2025). However, outcomes for patients who relapse have remained static at approximately 50%, making relapsed ALL one of the leading causes of death in childhood cancers (Sidhu et al., 2024).

Asparaginase or PEG-asparaginase (PEG-ASP) is a key drug in the treatment regimen for acute lymphoblastic leukemia (ALL). However, it is often associated with a range of adverse reactions, among which pancreatitis is one of the most serious. Studies have shown that discontinuation of asparaginase due to toxicity increases the risk of relapse and compromises overall prognosis (Wang et al., 2025), posing a significant threat to patients’ quality of life and treatment continuity.

The reported incidence of asparaginase-associated pancreatitis (AAP) varies across studies. In a study of 129 patients, the overall incidence was 14.7%, comprising 4.7% acute symptomatic pancreatitis and 10.1% acute chemical pancreatitis (Cong et al., 2018). A larger cohort study of 509 children with ALL reported a lower incidence of 6.1% (Yu et al., 2022). In a smaller study of 50 children, however, the incidence reached as high as 18% (Alvarez et al., 2000). These differences highlight the variability in the incidence of AAP across different populations and study designs.

Importantly, AAP is a significant contributor to treatment interruption and failure, potentially leading to disease relapse. Preventing AAP and ensuring treatment continuity are critical to maintaining the high cure rates achieved in childhood ALL. Nevertheless, the risk factors for AAP remain incompletely defined. Some research suggests that older age, high-risk stratification, and high doses of asparaginase may increase the likelihood of AAP (Chen et al., 2025; Lynggaard et al., 2023; Oparaji et al., 2017). In addition, abnormal laboratory indicators, such as decreased albumin and increased triglycerides, have also been associated with AAP (Wu et al., 2022). Genetic predispositions, particularly polymorphisms in the ULK2 and ABCC4 genes, have been linked to AAP risk (Wang et al., 2020; Bartram et al., 2021). Therefore, accurate identification and effective management of AAP risk factors, along with the development of reliable predictive models, are of significant clinical importance for maintaining treatment intensity, ensuring treatment continuity, and improving patient survival rates.

Although some studies have investigated AAP risk factors, no integrated predictive model for this adverse reaction currently exists. Developing such a model would allow for the comprehensive assessment of multiple interacting factors, enabling more accurate estimation of AAP risk in patients. This approach could provide a valuable tool for clinical decision-making by facilitating the early identification of high-risk individuals and supporting the implementation of targeted preventive measures. Accordingly, we aimed to systematically analyze the independent risk factors for AAP caused by PEG-ASP and construct a visual predictive model to provide evidence-based support for clinical practice.

## 2 Materials and methods

### 2.1 Study participants

This retrospective study included children newly diagnosed with ALL at Hebei Children’s Hospital from January 2020 to June 2025. All patients received standardized treatment according to the Chinese Children’s Leukemia Group Acute Lymphoblastic Leukemia 2018 (CCLG–ALL–2018) protocol. Patients were divided into AAP and non-AAP groups according to the 2012 Atlanta diagnostic criteria for pancreatitis.

### 2.2 Inclusion and exclusion criteria

#### 2.2.1 Inclusion criteria

(1) Children newly diagnosed with ALL at Hebei Children’s Hospital from January 2020 to June 2025 who received standardized treatment according to the CCLG–ALL–2018 protocol (National Health Commission of the People’s Republic of China, 2018); (2) patients who underwent complete diagnostic workup and subtype classification based on Morphology–Immunophenotype–Cytogenetics–Molecular Biology; (3) patients who met the diagnostic criteria for acute pancreatitis after asparaginase exposure (American Academy of Pediatrics).

Pancreatitis Diagnostic Criteria (Banks et al., 2013):

The 2012 Atlanta criteria require at least two of the following three characteristics for diagnosing acute pancreatitis: (1) acute, persistent, and severe upper abdominal pain; (2) serum lipase or amylase levels at least three times the upper limit of normal (ULN); and (3) imaging evidence of pancreatic enlargement, exudation, or necrosis through abdominal ultrasound, computed tomography, or magnetic resonance imaging.

#### 2.2.2 Exclusion criteria

(1) Children with incomplete baseline data or missing key clinical data during treatment; (2) children with pancreatitis due to trauma, biliary obstruction, or hyperlipidemia; and (3) children with a history of pancreatitis not related to asparaginase.

### 2.3 Data collection

The following variables were collected for analysis: (1) Basic characteristics: age, sex, body surface area, immunophenotyping, disease risk stratification, and central nervous system status; (2) Laboratory indicators: baseline indicators and biochemical indicators within 4 weeks after each PEG-ASP exposure (including albumin, transaminases, bilirubin, blood calcium, and blood glucose). For statistical analyses, the extreme value (maximum or minimum) of each indicator during this period was used. In this study, each patient was treated as an individual participant, and each administration of PEG-ASP was considered an independent exposure event.

### 2.4 Statistical methods

All statistical analyses and figure generation were performed using R 4.5.2 software, with extension packages including “rms,” “riskRegression,” “pROC”, “Hmisc”, “glmnet”, “nortest”, “dplyr”, “rmda” and “forestplot”. Statistical significance was set at a two-sided *P* < 0.05.

Continuous variables were first assessed for normality. Variables with a normal distribution were presented as mean ± standard deviation (x̄±s) and compared between groups using the t-test. Variables with a non-normal distribution were expressed as median (P25, P75) and compared using Mann–Whitney U test or Wilcoxon rank-sum test. Categorical variables were summarized as number (percentage) (n [%]) and compared between groups using the chi-square test.

Least absolute shrinkage and selection operator (LASSO) regression combined with cross-validation was used for variable selection, with the “Lambda.min” value—corresponding to the minimum cross-validated error—used as the optimal parameter to identify potential risk factors with non-zero coefficients. Based on the selected variables, a multivariate logistic regression model was constructed, and a nomogram was generated. Internal validation was performed using 1,000 bootstrap resamples. Model discrimination was evaluated using the receiver operating characteristic (ROC) curve and area under the curve (AUC); calibration was assessed using calibration curves to examine the agreement between predicted and observed outcomes; and clinical utility was evaluated using decision curve analysis (DCA).

## 3 Results

### 3.1 Basic characteristics of study participants

After applying the inclusion and exclusion criteria, 352 of 361 eligible patients were included in the final analysis. The cohort consisted of 199 boys (56.53%) and 153 girls (43.47%), with a median age of 5.0 years. Among the 352 patients, 36 (10.23%) developed AAP. Of these, 27 experienced one episode, 7 experienced two episodes, 1 experienced three episodes, and 1 experienced four episodes. No statistically significant differences in baseline characteristics were observed between the AAP group and the non-AAP group (*P* > 0.05; Table 1).

**TABLE 1:** Baseline data and characteristics (n = 352).

Variables	Overall (*n* = 352)	Non-AAP (*n* = 316)	AAP (*n* = 36)
Age (years)	5.0 (3.4, 8.0)	4.9 (3.4, 7.9)	5.5 (3.8, 8.4)
Body surface area (m^2^)	0.73 (0.60, 0.97)	0.72 (0.60, 0.96)	0.80 (0.67, 0.99)
Sex			
Male	199 (56.53)	179 (56.65)	20 (55.56)
Female	153 (43.47)	137 (43.35)	16 (44.44)
Central nervous system status			
CNS1	295 (83.81)	267 (84.49)	28 (77.78)
CNS2	9 (2.56)	7 (2.22)	2 (5.56)
CNS3	48 (13.64)	42 (13.29)	6 (16.67)
Immunophenotyping			
B-ALL	315 (89.49)	286 (90.51)	29 (80.56)
T-ALL	37 (10.51)	30 (9.49)	7 (19.44)
Risk group			
LR	51 (14.49)	48 (15.19)	3 (8.33)
IR	234 (66.48)	212 (67.09)	22 (61.11)
HR	67 (19.03)	56 (17.72)	11 (30.56)
Laboratory findings			
WBC (×10^9^/L)	8.9 (4.2, 30.3)	8.6 (4.0, 30.3)	10.9 (7.4, 41.1)
<50	290 (82.39)	263 (83.23)	27 (75.00)
≥50,<300	54 (15.34)	46 (14.56)	8 (22.22)
≥300	8 (2.27)	7 (2.22)	1 (2.78)
APTT (s)	26.8 (24.7, 29.1)	26.9 (24.6, 29.3)	26.6 (25.4, 28.8)
Fibrinogen (g/L)	3.02 (2.35, 4.06)	3.09 (2.35, 4.06)	2.73 (2.29, 4.16)
Albumin (g/L)	41.1 (38.5, 43.8)	41.1 (38.4, 43.6)	41.3 (39.1, 44.3)
Blood calcium (mmol/L)	2.35 (2.25, 2.44)	2.35 (2.24, 2.43)	2.35 (2.25, 2.45)
ALT (U/L)	13 (9, 23)	13 (9, 22)	13 (10, 26)
AST (U/L)	29 (21, 43)	29 (21, 42)	27 (20, 51)
LDH (U/L)	450 (284, 891)	452 (281, 913)	430 (317, 772)
TBIL (μmol/L)	5.60 (4.00, 8.70)	5.55 (3.90, 8.80)	5.90 (4.58, 7.90)
DBIL (μmol/L)	2.60 (1.90, 3.90)	2.60 (1.80, 3.90)	2.65 (2.10, 3.80)
Blood glucose (mmol/L)	5.33 (4.79, 5.84)	5.33 (4.81, 5.85)	5.35 (4.68, 5.69)

Abbreviations: B-ALL, B-cell acute lymphoblastic leukemia; T-ALL, T-cell acute lymphoblastic leukemia; LR, low risk; IR, intermediate risk; HR, high risk; WBC, white blood cell; APTT, activated partial thromboplastin time; ALT, alanine aminotransferase; AST, aspartate aminotransferase; LDH, lactic dehydrogenase; TBIL, total bilirubin; DBIL, direct bilirubin.

Variables with a non-normal distribution were expressed as median (P25, P75) and compared using the rank-sum test. Categorical variables were summarized as number (percentage) (n [%]) and compared between groups using the chi-square test. No significant difference was observed between the two groups.

Using each asparaginase exposure as a statistical unit, a total of 2,690 exposures occurred among the 352 patients, including 2,642 exposures without AAP and 48 exposures with AAP. Exposure-related characteristics for the two groups are detailed in Table 2.

**TABLE 2:** Characteristics after asparaginase exposure and variable assignments (n = 2,690 exposures).

Code	Variable and assignments	Non-AAP (*n* = 2,642)	AAP (*n* = 48)	*P*-value
X1	Sex	​	​	0.537
	‘Male’ = 1	1,534 (58.06)	30 (62.50)	​
	‘Female’ = 2	1,108 (41.94)	18 (37.50)	​
X2	Age (years)	5.5 (3.7, 8.7)	6.1 (4.2, 8.7)	0.425
X3	Body surface area (m^2^)	0.75 (0.61, 1.00)	0.85 (0.70, 1.00)	0.073
X4	Immunophenotyping	​	​	0.000***
	‘B-ALL’ = 1	2,304 (87.21)	33 (68.75)	​
	‘T-ALL’ = 2	338 (12.79)	15 (31.25)	​
X5	Risk group	​	​	0.279
	‘LR’ = 1	209 (7.91)	3 (6.25)	​
	‘IR’ = 2	1,764 (66.77)	28 (58.33)	​
	‘HR’ = 3	669 (25.32)	17 (35.42)	​
X6	Central nervous system status	​	​	0.313
	‘CNS1’ = 1	2,171 (82.17)	37 (77.08)	​
	‘CNS2’ = 2	72 (2.73)	3 (6.25)	​
	‘CNS3’ = 3	399 (15.10)	8 (16.67)	​
X7	Regimen	​	​	0.890
	‘VDLP/VDLD’ = 1	1,315 (49.77)	23 (47.92)	​
	‘CAML’ = 2	1,016 (38.46)	20 (41.67)	​
	‘HR-1’/‘HR-2’/‘HR-3’= 3	311 (11.77)	5 (10.42)	​
X8	Dose (IU/m^2^)	​	​	0.022*
	‘≤2000’ = 1	516 (19.53)	17 (35.42)	​
	‘>2000, <2,500’ = 2	1,845 (69.83)	26 (54.17)	​
	‘≥2,500’ = 3	281 (10.64)	5 (10.42)	​
X9	Dose number	​	​	0.371
	‘≤4’ = 1	1,328 (50.26)	25 (52.08)	​
	‘>4, ≤8’ = 2	1,020 (38.61)	15 (31.25)	​
	‘>8’ = 3	294 (11.13)	8 (16.67)	​
X10	History of AAP	​	​	0.000***
	‘No’ = 0	2,558 (96.82)	36 (75.00)	​
	‘Yes’ = 1	84 (3.18)	12 (25.00)	​
X11	Albumin (g/L)	36.1 (33.1, 38.9)	30.6 (28.2, 33.3)	0.000***
X12	ALT (U/L)	33 (21, 57)	62 (29, 119)	0.000***
X13	AST (U/L)	29 (20, 41)	46 (28, 112)	0.000***
X14	TBIL (μmol/L)	13.0 (8.1, 20.0)	19.0 (12.1, 31.0)	0.000***
X15	DBIL (μmol/L)	6.3 (4.1, 9.4)	9.5 (5.6, 15.7)	0.000***
X16	Blood calcium (mmol/L)	2.25 (2.18, 2.33)	2.08 (1.97, 2.19)	0.000***
X17	Blood glucose (mmol/L)	4.05 (3.57, 4.69)	6.65 (4.72, 8.22)	0.000***
X18	TG	​	​	0.092
	‘Normal’ = 0	2,472 (93.57)	42 (87.50)	​
	‘Abnormal’ = 1	170 (6.43)	6 (12.50)	​
Y	Pancreatitis	‘No’ = 0	‘Yes’ = 1	​

This table uses each PEG-asparaginase exposure as the unit of analysis; a total of 2,690 exposures are included.

Abbreviations: B-ALL, B-cell acute lymphoblastic leukemia; T-ALL, T-cell acute lymphoblastic leukemia; LR, low risk; IR, intermediate risk; HR, high risk; WBC, white blood cell; APTT, activated partial thromboplastin time; ALT, alanine aminotransferase; AST, aspartate aminotransferase; LDH, lactic dehydrogenase; TBIL, total bilirubin; DBIL, direct bilirubin; TG, triglycerides.

Variables with a non-normal distribution were expressed as median (P25, P75) and compared using the rank-sum test. Categorical variables were summarized as number (percentage) (n [%]) and compared between groups using the chi-square test.

Significance: *P < 0.05, **P < 0.01, ***P < 0.001.

### 3.2 Independent risk factors for AAP

LASSO regression identified 10 potential risk factors, including age, immunophenotyping, central nervous system status, asparaginase dosage, history of AAP (defined as AAP occurring during previous treatment courses with complete resolution before current PEG-ASP administration), albumin level, aspartate aminotransferase (AST), total bilirubin (TBIL), blood calcium, and blood glucose (Supplementary Figure S1). These variables were included in the multivariate logistic regression analysis, and ultimately, a history of AAP, albumin level, AST, TBIL, blood calcium, and blood glucose were determined to be independent risk factors for AAP (*P* < 0.05).

### 3.3 Nomogram prediction model construction and validation

#### 3.3.1 Model construction

**FIGURE 1:**
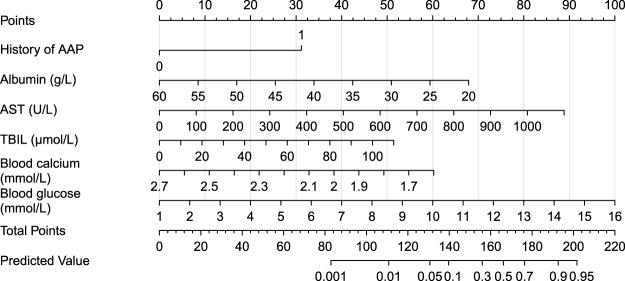
Nomogram for predicting the risk of asparaginase-associated pancreatitis (AAP). This figure integrates six independent risk factors, including a history of pancreatitis, albumin, aspartate aminotransferase, total bilirubin, blood calcium, and blood glucose. The total score is calculated by summing the scores corresponding to each indicator to predict the probability of AAP occurrence.

**FIGURE 2:**
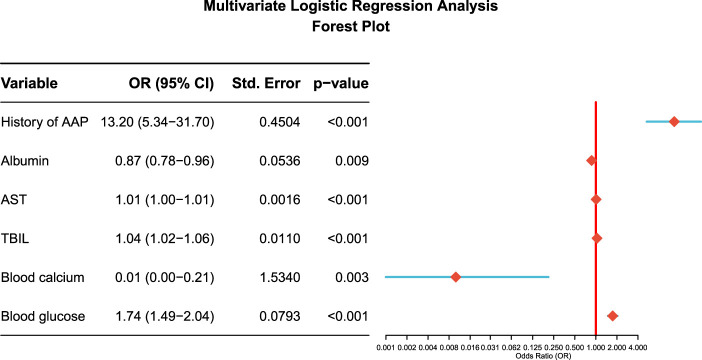
Forest plot of asparaginase-associated pancreatitis (AAP) risk factors. It intuitively displays the odds ratio (OR) and 95% confidence interval of each independent risk factor, clarifying the impact strength of each factor on AAP occurrence.

Based on the six independent risk factors confirmed by multivariate logistic regression analysis, a nomogram was constructed to predict the risk of AAP, allowing intuitive quantification of each patient’s probability of developing the condition (Figure 1). The odds ratios (OR) and 95% confidence intervals of each independent risk factor are shown in the forest plot (Figure 2).

#### 3.3.2 Model discrimination and calibration

**FIGURE 3:**
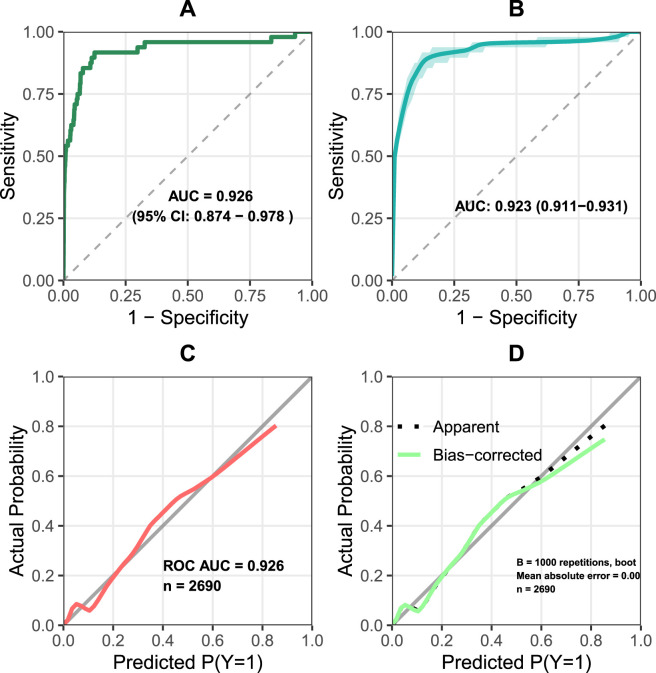
ROC and calibration curves of the prediction model **(A)** ROC curve of the model (AUC = 0.926) **(B)** ROC curve after 1,000 bootstrap validations (corrected AUC = 0.923) **(C)** calibration curve of the model **(D)** calibration curve after validation, demonstrating the model’s excellent discrimination and calibration.

The ROC curve for the predictive model yielded an AUC of 0.926 (95% CI: 0.874–0.978), indicating excellent discrimination. The calibration curve demonstrated good agreement between the nomogram-predicted AAP risk and the observed outcomes, with a Brier score of 0.012. Internal validation using 1,000 bootstrap resamples produced a corrected AUC of 0.923 (95% CI: 0.911–0.931), and the corresponding calibration curve also showed strong concordance between predicted and observed values (Figure 3).

#### 3.3.3 Model clinical applicability

**FIGURE 4:**
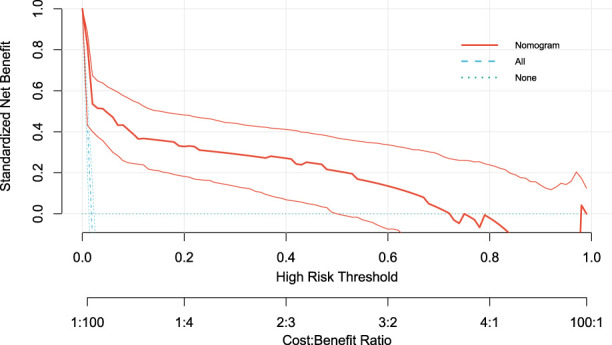
Decision curve analysis of the model. It shows that when the threshold probability is between 0.02 and 0.72, the model has a positive standardized net benefit, indicating that the model has clinical utility within this range.

The DCA results showed that when the threshold probability ranged from 0.02 to 0.72, the nomogram model provided a positive standardized net benefit, demonstrating optimal decision-making efficiency and indicating that the model is clinically useful within this range (Figure 4).

## 4 Discussion

This study retrospectively analyzed the clinical data of children with ALL treated according to the CCLG–ALL–2018 protocol, identifying a history of AAP, albumin level, AST, TBIL, blood calcium, and blood glucose as independent risk factors for AAP, and constructed a visual nomogram prediction model to provide a precise tool for early identification of high-risk children with AAP in clinical practice.

From a mechanistic perspective, a history of AAP has a particularly strong risk-doubling effect. A total of 36 patients developed AAP in this study, among whom nine had recurrent AAP (i.e., those who had previously experienced AAP during prior cycles of ALL treatment), yielding a recurrence rate of 25%. This indicates that prior pancreatic injury is a major risk factor for AAP. Consistent with our findings, a previous retrospective study (n = 7,640) reported that the recurrence rates of AAP after an asparaginase “rechallenge” were 20.8% in the low-risk group and 33.8% in the intermediate-to-high-risk group (Chen et al., 2025). Similarly, other studies reported a recurrence rate of 30.8% in patients rechallenged with asparaginase (Hassan et al., 2025). The mechanism of asparaginase-induced pancreatic injury is not yet fully understood. Some scholars have proposed that systemic asparagine depletion may reduce protein synthesis in high-metabolism organs, such as the pancreas and liver, leading to tissue damage (Wu et al., 2022). Other studies have shown that asparaginase can elevate pancreatic enzyme levels, including amylase and trypsin, directly causing histological damage to pancreatic acinar cells, indicating a direct toxic effect on the pancreas (Suzuki et al., 2008). Moreover, a history of pancreatitis may lead to pancreatic fibrosis and impaired acinar cell function, significantly reducing the pancreas’s tolerance to drug-induced toxicity and thereby increasing the risk of AAP recurrence.

Asparaginase is associated with liver function impairment, manifested as elevated transaminases, increased bilirubin, and reduced albumin synthesis (Zhou et al., 2016; Cong et al., 2018). In this study, the median albumin level in the AAP group (30.6 g/L) was significantly lower than that in the non-AAP group (36.1 g/L), consistent with the conclusions of Wu Ying et al. (Wu et al., 2022). Mechanistically, asparaginase degrades asparagine to inhibit tumor cell protein synthesis, but its nonspecific effects also affect normal cell metabolism, leading to decreased plasma albumin levels (Fu et al., 2007). In addition, the systemic inflammatory response associated with AAP can promote protein catabolism and increase capillary permeability through inflammatory mediators such as IL-6 and TNF-α, further exacerbating albumin loss (Amri et al., 2024; Cong et al., 2018). Albumin serves multiple functions, including maintaining colloid osmotic pressure, exerting anti-inflammatory and antioxidant effects, and protecting microvascular integrity. Reduced albumin levels not only reflect disease severity but may also contribute to persistent organ failure by impairing pancreatic microcirculation (Hong et al., 2017). AST and TBIL levels were significantly higher in the AAP group than in the non-AAP group, indicating a close association between liver function abnormalities and AAP. AST is widely distributed in multiple organs such as the liver, heart, and kidneys, and its elevation more accurately reflects the multi-organ damage caused by AAP. Elevated total bilirubin indicates hepatocellular injury or cholestasis, which indirectly interferes with pancreatic exocrine function by affecting bile secretion. Compared with direct bilirubin (DBIL), which is specific but less sensitive, total bilirubin provides a more comprehensive reflection of metabolic disturbances in the systemic inflammatory state of AAP and serves as an important indicator for predicting the risk of organ damage (Shao et al., 2025; Zhang et al., 2023).

Hypocalcemia is a characteristic pathological feature of AAP and aligns with the classic mechanism of pancreatitis, in which lipase activation leads to fat hydrolysis, the formation of calcium soaps from free fatty acids, and a subsequent decrease in serum calcium levels (D'Souza and Floch, 1973). Hypocalcemia is both an indicator of pancreatitis severity and an independent risk factor for poor prognosis (Ahmed et al., 2016), making it a useful marker for monitoring AAP. Hyperglycemia is a common manifestation in patients with AAP (Kuo et al., 2022). In this study, the median blood glucose level in the AAP group (6.65 mmol/L) was significantly higher than that in the non-AAP group (4.05 mmol/L). This elevation may be explained by two mechanisms: first, asparaginase impairs insulin function (CSCO et al., 2015); second, pancreatic tissue damage reduces insulin secretion by pancreatic β-cells (Wasserfall et al., 2025). Together, these effects lead to hyperglycemia, and severe cases may present with stress-induced hyperglycemia. Therefore, dynamically monitoring blood glucose levels is of great significance for early AAP identification in high-risk patients.

A systematic review of AAP risk factors in 2017 suggested that older age, asparaginase formulation, higher ALL risk stratification, and higher asparaginase dosing have a limited impact on AAP development (Lynggaard et al., 2023). By including a broader range of clinical indicators, the present study further clarified the predictive value of laboratory parameters, thereby addressing gaps in prior research.

Regarding age, most studies consider older children to be at higher risk for AAP (Chen et al., 2025; Oparaji et al., 2017; Lin et al., 2025), potentially due to insulin resistance, metabolic syndrome, dyslipidemia, and enhanced oxidative stress associated with hormonal changes during puberty (Agirbasli et al., 2009; Verlaan et al., 2006; Dimitrijevic-Sreckovic et al., 2007; Gerber et al., 2017). However, some studies have reported a higher incidence of AAP in children under 5 years old, reaching 40% (Alrasheedi et al., 2025). In the present study, no significant association between age and AAP was observed, which may be related to the concentrated age distribution of the sample, as 82.67% of participants were aged 1–10 years. Regarding asparaginase dosing, higher doses can increase toxicity and treatment interruptions, which may negatively affect long-term cure rates (Gibson A et al., 2021), whereas lower doses may reduce the risk of pancreatitis (Lynggaard et al., 2023). Liu et al. reported that children receiving asparaginase doses >3750 IU/m^2^ or ≥5 administrations had a 6-fold higher incidence of AAP than that of the low-dose group (Liu et al., 2016). Importantly, therapeutic levels can still be achieved in most patients even with reduced doses (Goldberg et al., 2024). Similarly, Xiaorong et al. reported that the incidence of AAP in the 2500 IU/m^2^ group was higher than that in the 2000 IU/m^2^group (Lai et al., 2024). In the present study, no significant association between dose and AAP risk was observed, which may be attributable to the center’s recent practice of reducing asparaginase doses to the lower limit of 2000 IU/m^2^ over the past 2 years. This dose standardization narrowed the dose gradient and lowered overall exposure, potentially diminishing the effect of dose variation on AAP incidence. Notably, the 2000 IU/m^2^ dose recommended by the CCCG–ALL–2015 protocol is associated with good long-term prognosis and lower treatment-related mortality (Chinese Children′s Cancer Group Acute Lymphoblastic Leukemia, 2015 Study Group, 2022), suggesting that within the standard dose range, individual patient risk factors may play a more prominent role in AAP development.

The nomogram constructed in this study provides an intuitive and reliable risk assessment tool for clinical practice. Based on the research results, a comprehensive, risk-stratified management strategy is recommended for the prevention of AAP. Prior to each asparaginase exposure, the nomogram model should be used to assess key risk factors, including a history of pancreatitis, albumin, AST, total bilirubin, blood calcium, and blood glucose levels, to identify high AAP risk patients. Routine monitoring of serum amylase, lipase, liver and kidney function, electrolytes, and blood glucose should be performed before and after medication. If symptoms such as abdominal pain and nausea occur, timely imaging studies should be performed to enable early diagnosis. For high AAP risk patients, asparaginase activity monitoring can guide individualized dose adjustments when feasible. Concurrently, hypoproteinemia, hypocalcemia, and hyperglycemia should be actively corrected through nutritional support, calcium supplementation, and glycemic control interventions. The risk of re-exposure should be carefully evaluated in patients with a history of pancreatitis. For patients who have experienced AAP, a multidisciplinary assessment should determine whether asparaginase therapy can continue. In children with intermediate-risk and high-risk ALL, complete discontinuation is not recommended unless life-threatening AAP occurs; dose reduction may be considered (Jia et al., 2025). Finally, a multidisciplinary collaboration mechanism involving hematology, gastroenterology, and nutrition departments should be established. Health education for patients and caregivers should be strengthened to increase awareness of early AAP symptoms and improve adherence to medical recommendations.

The current treatment strategy for ALL is increasingly shifting toward reducing chemotherapy intensity through the introduction of immunotherapy and targeted therapies to minimize toxicity and adverse effects (Torrent et al., 2024; Kantarjian et al., 2025). Novel immunotherapies such as blinatumomab provides strong support for further chemotherapy de-escalation, optimizing efficacy while improving quality of life, particularly for patients with low risk ALL (Inaba et al., 2019; Short et al., 2023). Investigators have also evaluated frontline and later-line regimens combining tyrosine kinase inhibitors and immunotherapies with reduced or no chemotherapy (Kantarjian et al., 2025).

The clinical value of this predictive model extends beyond individual patient diagnosis and treatment to encompass a comprehensive framework for pediatric ALL management. Currently, the primary challenge in pediatric ALL has shifted from achieving initial remission to preventing treatment-related complications that may lead to relapse. The occurrence of acute adverse events (AAEs) may compromise cure rates due to treatment discontinuation or premature asparaginase withdrawal and may potentially reduce survival rates in patients with relapsed ALL. By enabling early identification of high-risk AAE patients, our nomogram facilitates timely interventions to maintain treatment continuity, thereby safeguarding the high cure rates achieved with modern ALL treatment regimens. This approach employs individualized strategies to optimize therapeutic outcomes. With the continuous emergence of novel treatment modalities, maintaining the integrity of first-line chemotherapy regimens through effective toxicity prevention remains critical. The model provides practical and easily implementable tools to minimize treatment failures caused by AAEs, ultimately supporting the goal of achieving cure in more pediatric patients with ALL and contributing to this comprehensive therapeutic strategy.

This study has some limitations. First, as a single-center retrospective study with a limited sample size, selection bias may have been introduced. External validation in multicenter cohorts is required to confirm the model’s generalizability. The identified risk factors and the constructed predictive model therefore require further validation in multi-center, large-sample studies. Second, potential risk factors such as genetic polymorphisms and drug-metabolizing enzyme activities were not included, indicating that the model could be further refined by incorporating additional dimensions.

## Data Availability

The raw data supporting the conclusions of this article will be made available by the authors, without undue reservation.
