# Supplementary material for: Nomogram-based prediction of asparaginase-associated pancreatitis in children with acute lymphoblastic leukemia: a retrospective study
Source: Front Pharmacol. 2026 May 19;17:1767417. doi: 10.3389/fphar.2026.1767417 (PMC13226028; doi:10.3389/fphar.2026.1767417)
Supplement: Supplementary file 1 [file Supplementaryfile1.docx]

Supplementary Material

## Supplementary Figures


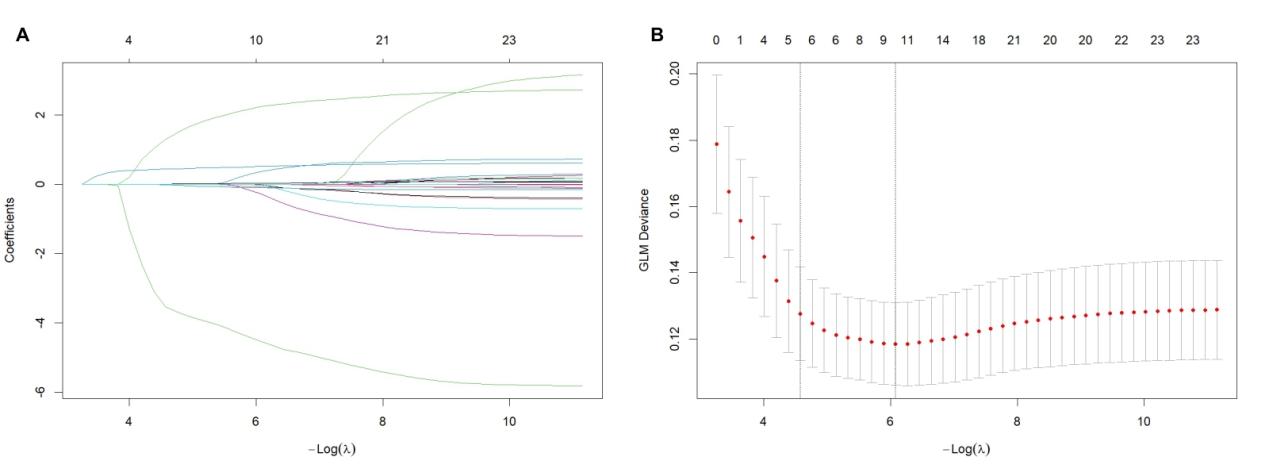


Supplementary Figure 1: LASSO regression for variable selection of asparaginase-induced pancreatitis factors. (**A**) Changes in LASSO coefficients of variables; (B) Selection process of the optimal parameter (λ) in the LASSO model by cross-validation with the minimum standard.

Supplementary Figure 1: LASSO regression variable selection. (**A**) Trajectory of variable coefficients with changing λ values; (**B**) Process of determining the optimal λ value (Lambda.min) by cross-validation, ultimately selecting 10 potential risk factors.
